# Effects of Perioperative Magnesium Sulfate Administration on Postoperative Chronic Knee Pain in Patients Undergoing Total Knee Arthroplasty: A Retrospective Evaluation

**DOI:** 10.3390/jcm8122231

**Published:** 2019-12-17

**Authors:** Tak Kyu Oh, Seung Hyun Chung, Jinwoo Park, Hyunjung Shin, Chong Bum Chang, Tae Kyun Kim, Sang-Hwan Do

**Affiliations:** 1 Department of Anesthesiology and Pain Medicine, Seoul National University Bundang Hospital, Seongnam 13620, Korea; airohtak@hotmail.com (T.K.O.); jinul8282@snubh.org (J.P.); medidoc@nate.com (H.S.); 2 Department of Orthopedic Surgery, Seoul National University Bundang Hospital, Seongnam 13620, Korea; ccbknee@snubh.org; 3 TK Orthopedic Surgery, Seongnam 13533, Korea; osktk2000@yahoo.com; 4 Department of Anesthesiology and Pain Medicine, College of Medicine, Seoul National University, Seoul 03080, Korea

**Keywords:** analgesia, spinal anesthesia, total knee arthroplasty, magnesium sulfate, chronic pain

## Abstract

We aimed to investigate whether perioperative magnesium sulfate administration was associated with the incidence of chronic persistent postoperative pain (PPP) following total knee arthroplasty (TKA). This retrospective observational study was performed at a single tertiary academic hospital. We reviewed the medical records of adult patients who were admitted between August 2012 and July 2017. Patients who received magnesium sulfate during surgery were the magnesium group. The presence of PPP, one year after TKA, was evaluated using a binary logistic regression analysis. A total of 924 patients were included in the analysis, and 148 patients (16.0%) experienced PPP one year after TKA. In the multivariable model, the magnesium group had a 62% lower rate of PPP one year after TKA compared to the control group (odds ratio (OR): 0.38, 95% confidence interval (CI): 0.16 to 0.90; *p* = 0.027). This finding was similar in the sensitivity analysis using propensity score adjustment (OR: 0.38, 95% CI: 0.16 to 0.93; *p* = 0.036). We showed that perioperative magnesium sulfate administration was associated with a lower rate of PPP one year after TKA. Our results suggest that magnesium sulfate administered perioperatively is effective for the alleviation of acute and chronic pain after surgery.

## 1. Introduction

Total knee arthroplasty (TKA) is one of the most commonly performed surgical procedures in developed countries [1]. The primary purposes of TKA are pain relief and improvement in mobility and health-related quality of life in patients with advanced knee osteoarthritis (OA) [2]. Among the patients who undergo TKA, approximately 20% are reported to experience chronic persistent postoperative pain (PPP) [3,4]. Due to the expected future increase in the demand and number of TKAs performed [5], adverse outcomes—such as chronic PPP after TKA—should be of significant concern to patients, healthcare providers, and policy-makers [6,7].

Magnesium sulfate is an adjuvant drug that is administered during the perioperative period [8], and one of the benefits of its perioperative administration is the improvement of acute postoperative pain [9]. The analgesia-potentiating effect of magnesium sulfate is well-known in patients receiving a variety of surgeries including TKAs [10]. Furthermore, in addition to alleviating acute postoperative pain, magnesium sulfate may be effective at preventing chronic PPP after TKA. This effect is explained by two theories. First, the mechanism of the analgesic effect of magnesium sulfate is thought to be its antagonistic effect on N-methyl-D-aspartate (NMDA) receptors [11], which are associated with the development of central sensitization after peripheral tissue injury or inflammation [12,13]. Central sensitization is known to be an important mechanism of chronic PPP development, as described in a previous study [14]. Secondly, high intracellular calcium levels play a pivotal role in the initiation of central sensitization [15], and magnesium is a natural calcium channel antagonist. Therefore, the antagonizing effect of magnesium sulfate on calcium channels also attenuates both the development of central sensitization and chronic PPP after TKA. However, clinical studies on this aspect are currently lacking.

Therefore, this study aimed to investigate whether perioperative magnesium sulfate administration is associated with the incidence of chronic PPP following TKA. We hypothesized that perioperative magnesium sulfate administration will have the benefit of lowering the incidence of chronic PPP following TKA.

## 2. Materials and Methods

### 2.1. Ethical Statement

This study was a retrospective observational study at a single tertiary academic hospital, performed with approval by the Institutional Review Board (IRB) of Seoul National University Bundang Hospital (SNUBH) (Approval Number: B-1901/514-117, approval date: 24 December 2018). The IRB waived the requirement to obtain informed consent from the patients, because of the retrospective cohort design, which analyzed the health records of patients who had already completed their treatment. All medical records were collected anonymously by a medical record technician in the medical informatic team at SNUBH. In addition, this study was conducted according to the guidelines of Strengthening the Reporting of Observational Studies in Epidemiology (STROBE).

### 2.2. Study Population

The medical records of patients with primary OA who were 18 years or older and admitted between August 2012 and July 2017 for TKA under spinal anesthesia were included and analyzed. Patients with any of the following criteria were excluded from the study: (1) preoperative American Society of Anesthesiologists (ASA) physical status ≥3; (2) TKA for secondary OA; (3) history of previous surgery on the knee; (4) failure of femoral nerve catheterization due to leakage, occlusion, or accidental dislodgement of the catheter; (5) epidural patient controlled analgesia (PCA) received perioperatively; (6) loss to follow-up for the evaluation of chronic PPP one year after TKA. If a patient received a staged TKA within one week, only the information from the first TKA surgery was included in the analysis.

### 2.3. Perioperative Care for Patients Undergoing TKA

As reported in our previous studies [16,17], anesthetists administered spinal anesthesia using a hyperbaric bupivacaine (Marcaine^®^ Spinal 0.5% Heavy, AstraZeneca, Cambridge, UK) and 10 or 15 µg fentanyl for a unilateral TKA. The targeted sensory block level was T10, and after confirming the correct block level, sedation was performed during the surgery using propofol or dexmedetomidine. Following the TKA procedure, a periarticular injection containing a total of 300 mg of ropivacaine, 10 mg of morphine, 30 mg of ketorolac, 300 μg of 1:1000 epinephrine, and 750 mg of cefuroxime was administered in divided doses into the sheath of the medial and lateral collateral ligaments, the posterior capsule, synovium, capsule, quadriceps muscle, subcutaneous tissue, and joint capsule [18]. Postoperative pain management consisted of the administration of fentanyl-based intravenous (IV) PCA pumps. The regimen, based on the patient’s weight and any underlying diseases, was as follows: fentanyl 8–15 µg/mL, 1 mL bolus dose without a background infusion, and a 10-min lockout time. IV-PCA (100 mL total) was primarily used on postoperative days 0–3. Additionally, patients were prescribed 650 mg of oral acetaminophen, 200 mg of celecoxib, and 75 mg of pregabalin every 12 h after the TKA for postoperative pain control. Rescue opioid analgesics (morphine, oxycodone, or tramadol) and antiemetics, including 50 mg of IV ketoprofen and 10 mg of metoclopramide, respectively, were administered upon patients’ requests. For adjuvant pain control, a femoral nerve block was administered by the anesthetist, with 0.2% ropivacaine infused through a catheter at a rate of 4 mL per hour, from the start of the surgery until 48 h after the operation.

### 2.4. Surgical Procedures for TKA

We included only patients who underwent surgery performed by the same orthopedic surgeon (TK Kim) to ensure consistency in the operative technique and procedure used in all participants. The surgical team led by TK Kim routinely performed the partial resection of the part of the infrapatellar fat pad (IFP) that was thought to obstruct clear visualization and/or get trapped in the joint.

### 2.5. Exposure Variable: Perioperative Magnesium Sulfate Administration

In SNUBH, magnesium sulfate infusion in spinal anesthesia was used routinely by S. H. Do (principal investigator of this study) for TKA, with the intention to improve postoperative pain control and prolong the duration of the spinal sensorial block [10,19]. In the operating room, a magnesium sulfate infusion was started with the induction of spinal anesthesia until the end of the surgery. A mixture of 50 mg/kg of magnesium sulfate in 100 mL of isotonic saline was infused over 15 min during the induction of anesthesia, and the infusion rate was adjusted throughout the surgery using the reference rate of 15 mg/kg/h based on the patient’s vital signs. If there was a complication due to the magnesium sulfate infusion during surgery, such as hypotension, the anesthetist administered additional intravenous fluids and/or a vasopressor. The patients who received magnesium sulfate during surgery were defined as the magnesium group, and the other patients were defined as the control group. If magnesium sulfate was administered during the first surgery in patients who received a staged TKA within one week, magnesium sulfate was given during the succeeding surgeries as well. Similarly, if patients did not receive magnesium sulfate during the first TKA surgery, magnesium sulfate was not administered during the succeeding surgeries.

### 2.6. Primary Endpoint: Presence of Chronic PPP One Year after TKA

Chronic PPP one year after TKA was the primary endpoint of this study. The presence of chronic PPP was defined 12 months after TKA in 2 ways: (1) persistent pain in the operated knee; (2) the patient was prescribed analgesics for pain in the operated knee. These cases of chronic PPP were identified in a review of medical records that took place 11 to 13 months after TKA was performed. If a patient received a staged TKA within one week, chronic PPP was measured based on the date of the first TKA surgery.

### 2.7. Baseline Characteristics and Confounding Parameters

The following data were collected from the medical records of patients: sex, age, body mass index (kg/m^2^), type of surgery (unilateral TKA/staged TKA/bilateral TKA), magnesium sulfate use, preoperative ASA physical status, duration of surgery and anesthesia, intraoperative sedation (none/propofol/dexmedetomidine), and perioperative midazolam use (mg).

### 2.8. Statistical Analysis

The baseline characteristics of the patients were presented as numbers with the percentages for categorical variables and as the mean with standard deviations for continuous variables. First, we performed uni- and multivariable binary logistic regression for PPP one year after surgery in patients who underwent TKA. In the binary logistic regression analysis, the presence of chronic PPP one year after surgery was the dependent variable. Thereafter, all covariates were included in a multivariable model for adjustment, except for the duration of anesthesia in order to avoid multi-collinearity. The goodness of fit for the multivariable model was tested using the Hosmer–Lemeshow test.

To enhance the robustness of our main findings, we performed propensity score (PS) matching, which is known to reduce confounders in observational studies [20]. For the PS matching, the nearest neighbor method was used without replacement, using a 1:3 ratio within a caliper width of 0.2. All covariates were included in the PS model, and a logistic regression analysis was performed to calculate the PS as a logistic model. The absolute value of the standardized mean difference (ASD) was used to evaluate the balance between the magnesium and control groups before and after the PS matching. The ASD was set at <0.1 to confirm the balance between the two groups. The results of the logistic regression models were presented as odds ratios (ORs) with 95% confidence intervals (CIs). All statistical analyses were performed using the R software (version 3.6.1 with R packages; R Foundation for Statistical Computing, Vienna, Austria), and a *p* < 0.05 was considered statistically significant.

## 3. Results

**Figure 1:**
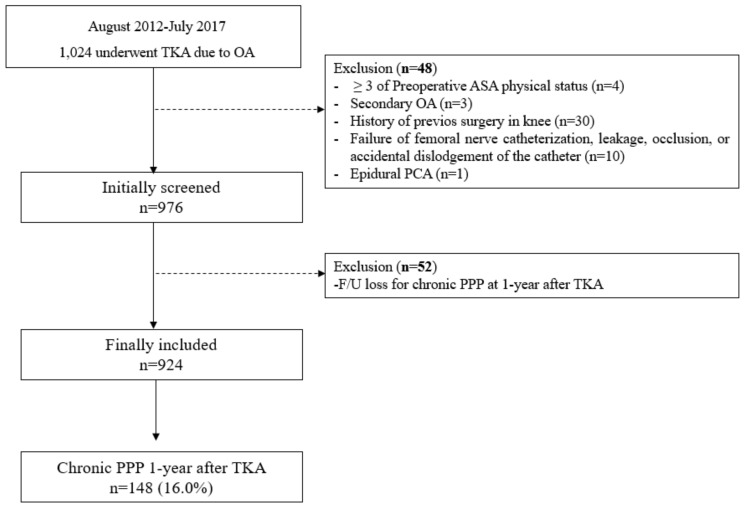
Flow chart depicting patient selection. TKA, total knee arthroplasty; OA, osteoarthritis; ASA, American Society of Anesthesiologists; PCA, patient controlled analgesia; F/U, follow-up; PPP, persistent postoperative pain.

From August 2012 to July 2017, 1024 patients underwent TKA due to primary OA. Among them, 48 patients were excluded for the following reasons: preoperative ASA physical status ≥ 3 (*n* = 4), secondary OA (*n* = 3), history of previous surgery on the knee (*n* = 30), failed femoral nerve block (*n* = 10), and epidural PCA administered (*n* = 1). A further 52 patients were excluded because they were lost to follow-up for the evaluation of PPP one year after TKA. Therefore, a total of 924 patients were eventually included in the analysis, and 148 patients (16.0%) had PPP one year after TKA (Figure 1). The baseline characteristics of all patients are presented in Table 1.

### 3.1. Uni- and Multivariable Logistic Regression Analysis

Table 2 shows the results of the uni- and multivariable logistic regression analysis for PPP one year after TKA. In the univariable model, the reduction in the rate of PPP one year after TKA was found to be 65% (OR: 0.35, 95% CI: 0.15 to 0.81; *p* = 0.015). In the multivariable model, the reduction in the rate of PPP one year after TKA was also found to be 62% (OR: 0.38, 95% CI: 0.16 to 0.90; *p* = 0.027) in the magnesium and control groups. Among other factors, a staged TKA was associated with a 2.06-fold higher rate of PPP one year after TKA, compared to that following a unilateral TKA (OR: 2.06, 95% CI: 1.40 to 3.02; *p* < 0.001).

### 3.2. Sensitivity Analysis after PS Adjustment

Table S1 shows the results of the comparison between the magnesium and control groups before and after PS matching, and Table 3 shows the results of the sensitivity analysis in the PS-matched cohorts. After PS adjustment, 6.7% (6 of 89) of patients in the magnesium group had PPP one year after TKA, while 15.9% (37 of 232) of patients in the control group had PPP one year after TKA. In the logistic regression analysis, the magnesium group had a 62% lower incidence of PPP one year after TKA compared to that in the control group (OR: 0.38, 95% CI: 0.16 to 0.93; *p* = 0.036)

## 4. Discussion

This retrospective study showed that perioperative magnesium sulfate administration was associated with a lower incidence of PPP one year after TKA. The results were confirmed by PS adjustment and suggested that perioperative magnesium sulfate administration has a benefit with regard to chronic PPP one year after TKA. In this study, we investigated PPP one year after TKA because patients were more likely to be seen by the surgeon at that time than 3 or 6 months after TKA in our institution. In this study, the proportion of patients with PPP one year after TKA was 16.0%, which was exactly the same as that reported in a recent prospective cohort study [3].

In a previous meta-analysis, perioperative systemic magnesium sulfate administration reduced both postoperative pain and opioid administration [9]. This phenomenon was explained by the observation that magnesium sulfate inhibits calcium entry into cells by blocking NMDA receptors, which might cause an anti-nociceptive effect for acute painful stimuli [13]. Based on this rationale, we hypothesized that the magnesium group in this retrospective observational study might have less postoperative pain after TKA than the control group, as demonstrated in our previous randomized clinical trial [10]. Considering that perioperative pain is known to be a potentially modifiable predictive factor for the development of chronic PPP after TKA [21], perioperative magnesium sulfate administration might affect the results in our study.

The effect of magnesium sulfate administration on central sensitization also played an important role in this study. Acute postoperative pain is known to lead to central sensitization, which reduces the mechanical pain threshold and exaggerates the response to noxious stimuli [22]. Peripheral and central sensitization, which enhance the post-incisional pain sensation for a given nociceptive input level, are relevant risk factors for developing chronic PPP [12]. First, magnesium sulfate is known to antagonize NMDA receptors. The inhibition of dorsal horn NMDA receptors is known to attenuate peripheral and central sensitization after peripheral tissue injury or inflammation [12,13]. Second, magnesium sulfate inhibits calcium influx into cells via voltage-gated channels [15]. This phenomenon is associated with the anti-nociceptive effect of magnesium sulfate [23]. Considering that high intracellular calcium levels play an important role in the mechanism of central sensitization [24], magnesium sulfate may have attenuated the central sensitization after TKA in the magnesium group in our study.

Our study notably included cases of staged TKAs in the analysis. If magnesium sulfate was administered during the initial surgery in patients who received a staged TKA within one week, magnesium sulfate was given during the succeeding surgeries as well. Previous studies reported that the postoperative pain was more severe after the second TKA rather than in the first TKA surgery among patients who received staged TKAs [10,25]. Thus, the chronic PPP experienced by these patients may be challenging to manage in clinical practice. This study showed that magnesium sulfate infusions may be useful at preventing chronic PPP in patients who received staged TKAs.

A recent prospective observational study by Ghezel-Ahmadi et al. [26] reported that perioperative systemic magnesium sulfate administration reduced chronic post-thoracotomy pain, which was defined as PPP 90 days after thoracic surgery. The authors focused on chronic PPP after thoracic surgery, and their main finding was similar to that of our study. Because perioperative magnesium sulfate administration has analgesic effects in patients undergoing TKA under spinal anesthesia [10,27], future prospective and randomized clinical studies are required to investigate whether it also provides benefits for preventing chronic PPP after TKA. Our study provides important indicators for the calculation of the optimal sample size for such future clinical trials.

Issues regarding surgical technique should be considered in this study. We included patients who underwent TKA performed by one orthopedic surgeon (TK Kim) to ensure unity of surgical technique. Recently, the IFP has been investigated as a potential pain source of OA, and inflammation of the IFP might drive the peripheral and central sensitization that are associated with the transition from acute pain in the knee to chronic PPP in OA patients [28]. However, the effect of IFP resection in TKA for OA patients remains controversial in the current literature [29]. In this study, the surgical team led by TK Kim removed some of the IFP without exception, which might not have affected the results of our study.

There are a few limitations to this study. First, the retrospective design may result in potential selection bias; therefore, the accuracy and quality of the data might be inferior to prospectively collected data. Second, because this study was conducted at a single tertiary academic hospital, the results cannot be generalized as they might not be directly applicable to other institutions. Third, because the prevalence of end-stage OA was higher in female patients, only 7.5% of our study cohort were male. Therefore, it is not possible to generalize our findings to male patients. Fourth, the multivariable adjustment could only control for the known confounders in this retrospective observational study. There might be unknown confounders that affected the results of this study. Lastly, we did not include the history of addiction or personality disorder, which are known to affect prescription of opioid analgesics [30], and could influence our result.

## 5. Conclusions

This retrospective observational study showed that perioperative magnesium sulfate administration was associated with a lower rate of PPP one year after TKA, performed under spinal anesthesia. Our findings should be confirmed in prospective clinical trials.

## Figures and Tables

**Table 1:** Baseline characteristics of patients who received TKA.

Variable	Total 924 Patients (%)	Mean (SD)
Sex, female	855 (92.5)	
Age, year		71.7 (6.1)
Body mass index, kg/m^2^		27.0 (3.4)
Type of surgery		
Unilateral TKA	467 (50.5)	
Staged unilateral TKA	428 (46.3)	
Bilateral TKA	29 (3.1)	
Intraoperative magnesium sulfate infusion	90 (9.7)	
Preoperative ASA physical status		
1	121 (13.1)	
2	803 (86.9)	
Duration of surgery, min	99.8 (23.3)	
Duration of anesthesia. min	143.0 (27.5)	
Intraoperative sedation		
None	632 (68.4)	
Propofol	186 (20.1)	
Dexmedetomidine	106 (11.5)	
Premedication (*n* = 320, midazolam, mg)		2.5 (0.8)

SD, standard deviation; TKA, total knee arthroplasty; ASA, American Society of Anesthesiologists.

**Table 2:** Uni- and multivariable logistic regression analysis for chronic persistent postoperative pain one year after TKA.

Variable	Univariable Model	*p*-Value	Multivariable Model	*p*-Value
	OR (95% CI)		OR (95% CI)	
Sex, male	0.77 (0.38, 1.59)	0.485	0.92 (0.44, 1.94)	0.832
Age, year	0.99 (0.96, 1.02)	0.583	0.99 (0.96, 1.02)	0.430
Body mass index, kg/m^2^	1.02 (0.97, 1.08)	0.383	1.00 (0.95, 1.05)	0.948
Type of surgery				
Unilateral TKA	1		1	
Staged unilateral TKA	2.08 (1.44, 3.01)	<0.001	2.06 (1.40, 3.02)	<0.001
Bilateral TKA	1.63 (0.60, 4.45)	0.342	1.35 (0.26, 6.96)	0.722
Magnesium group	0.35 (0.15, 0.81)	0.015	0.38 (0.16, 0.90)	0.027
Preoperative ASA physical status				
1	1		1	
≥2	1.53 (0.85, 2.75)	0.155	1.43 (0.78, 2.61)	0.245
Duration of surgery, min	1.00 (1.00, 1.01)	0.250	1.00 (0.99, 1.01)	0.726
Duration of anesthesia. min	1.00 (0.99, 1.01)	0.449		
Intraoperative sedation				
None	1		1	
Propofol	1.11 (0.71, 1.71)	0.653	1.06 (0.68, 1.66)	0.796
Dexmedetomidine	0.95 (0.53, 1.68)	0.849	1.11 (0.60, 2.03)	0.746
Premedication (midazolam, mg)	1.10 (0.88, 1.37)	0.398	1.09 (0.87, 1.36)	0.466

Hosmer and Lemeshow Test, Chi-square: 5.66 (*p* = 0.686). Duration of anesthesia was not included in multivariable model to avoid multi-collinearity. TKA, total knee arthroplasty; OR, odds ratio; CI, confidence interval; ASA, American Society of Anesthesiologists.

**Table 3:** Sensitivity analysis in propensity score matched cohort.

Variable		CPPP One Year After TKA	Logistic Model	*p*-Value
		Event (%)	OR (95% CI)	
Unadjusted				
	Control group	142/834 (17.0)	1	
	Magnesium group	6/90 (6.7)	0.35 (0.15, 0.81)	0.015
After PS adjustment				
	Control group	37/232 (15.9)	1	
	Magnesium group	6/89 (6.7)	0.38 (0.16, 0.93)	0.036

CPPP, chronic persistent postoperative pain; TKA, total knee arthroplasty; OR, odds ratio; CI, confidence interval; PS, propensity score.

## Supplementary Materials

The following are available online at https://www.mdpi.com/2077-0383/8/12/2231/s1, Table S1: Characteristics between magnesium group and control group before and after PS matching.
