# Supplementary material for: Effects of Perioperative Magnesium Sulfate Administration on Postoperative Chronic Knee Pain in Patients Undergoing Total Knee Arthroplasty: A Retrospective Evaluation
Source: J Clin Med. 2019 Dec 17;8(12):2231. doi: 10.3390/jcm8122231 (PMC6947579; doi:10.3390/jcm8122231)
Supplement: Supplementary file 1 [file jcm-08-02231-s001.pdf]

Table S1. Characteristics between magnesium group and control group before and after PS matching

Variables	Entire cohort (n=924)			PS-matched cohort (n=321)		
	Mg group n=90	Control n=834	ASD	Mg group n=89	Control n=232	ASD
Age, year	73.6 (6.1)	71.5 (6.1)	0.349	73.6 (6.1)	72.9 (5.8)	0.099
Sex: male	60 (7.2)	9 (10.0)	0.093	9 (10.1)	22 (9.5)	0.019
Body mass index, kg/m^2	26.9 (3.6)	27.0 (3.3)	0.036	26.9 (3.6)	26.8 (3.3)	0.006
Type of surgery			0.168			0.065
Unilateral TKA	417 (50.0)	50 (55.6)		50 (56.2)	124 (53.4)	
Staged unilateral TKA	393 (47.1)	35 (38.9)		35 (39.3)	102 (44.0)	
Bilateral TKA	24 (2.9)	5 (5.6)		4 (4.5)	6 (2.6)	
Preoperative ASA physical status			0.070			0.077
1	10 (11.1)	111 (13.3)		10 (11.2)	33 (14.2)	
≥ 2	80 (88.9)	723 (86.7)		79 (88.8)	199 (85.8)	
Duration of surgery, min	100.9 (27.4)	99.7 (22.8)	0.046	99.9 (25.9)	98.8 (24.0)	0.018
Duration of anaesthesia, min	143.3 (27.4)	139.8 (28.8)	0.121	138.9 (27.5)	137.4 (27.1)	0.014
Intraoperative sedation			0.701			0.056

None	56 (62.2)	576 (69.1)		56 (62.9)	161 (69.4)	
Propofol	5 (5.6)	181 (21.7)		5 (5.6)	18 (7.8)	
Dexmedetomidine	29 (32.2)	77 (9.2)		28 (31.5)	53 (22.8)	
Premedication (midazolam, mg)	2.2 (1.0)	2.5 (0.8)	0.305	2.2 (1.0)	2.3 (1.0)	0.016

Data are presented as number (percentage) or mean (standard deviation).

ASD, absolute value of standardised mean difference; TKA, total knee arthroplasty; ASA, American Society of Anesthesiologist
